# New Insights into the Relationship Between Microplastics and Diabetes from the Perspective of the Gut–Liver Axis and Macrophage Regulation

**DOI:** 10.3390/toxics14030241

**Published:** 2026-03-10

**Authors:** Huasen Wang, Ben Liu, Xiangfeng Zhao

**Affiliations:** 1 Department of Immunology, Guilin Medical University, Guilin 541199, China; 2 School of Public Health, Quanzhou Medical College, Quanzhou 362011, China

**Keywords:** microplastics, type 2 diabetes, gut–liver axis, macrophage

## Abstract

Microplastics (MPs) are increasingly recognized as a global environmental threat. Emerging evidence suggests they may have metabolic consequences. In this review, we synthesize current findings from animal and in vitro studies to propose a mechanistic framework linking MP exposure to type 2 diabetes mellitus (T2DM). This framework is uniquely centered on the gut–liver axis and macrophage-centric immune networks. We systematically delineate evidence suggesting that MPs can compromise intestinal barrier integrity, instigate gut dysbiosis, and promote pro-inflammatory M1 polarization of macrophages in experimental models. This immune activation is proposed to subsequently amplify hepatic inflammation, potentially contributing to systemic insulin resistance (IR) and pancreatic β-cell dysfunction. We emphasize that while this pathway is biologically plausible, direct causal evidence in humans remains limited and is a critical knowledge gap. Integrating multi-level evidence from animal models and in vitro systems, we delve into the trans-organ immunometabolic effects of MPs within adipose tissue, pancreas, and skeletal muscle, establishing their role as a novel class of “metabolic disruptors.” Critically, we assess the key controversies and knowledge gaps pertaining to dose–response relationships, particle-specific toxicity (size, polymer type, and additives), the effects of complex environmental mixtures, and the urgent need for robust human validation. We advocate for future research priorities, including multi-omics integration, advanced organ-on-a-chip platforms, prospective cohort studies, and targeted intervention strategies, to propel this field from mechanistic exploration toward clinical and public health relevance. Finally, this synthesis underscores that mitigating the production and environmental release of MPs, alongside developing strategies to impede their bioavailability and accumulation, represents a crucial public health imperative for the prevention of environment-related metabolic diseases.

## 1. Background

The potential link between microplastics (MPs), a pervasive emerging environmental contaminant, and the rising prevalence of type 2 diabetes mellitus (T2DM) has become a research hotspot at the intersection of environmental medicine and metabolomics [1]. First identified in the ocean in 1972 [2], MPs are now omnipresent in water, air, and food, creating a multi-route human exposure landscape. Global plastic production reached 580 million tons in 2025, with an annual growth rate of 2.9%; at this trajectory, plastic waste accumulation is projected to reach approximately 120 billion tons by 2050 [3]. Humans are exposed via ingestion, inhalation, and dermal contact. Adults ingest an estimated 4.9–5.8 × 10^5^ MP particles annually through diet, with additional intake of 0.2–1.2 × 10^6^ and 0.2–2.5 × 10^6^ particles via drinking water and inhalation, respectively. Infants fed with plastic bottles ingest far higher amounts, making them a high-risk group [4,5]. Smaller MPs (nanoplastics < 0.1 μm) more readily cross biological barriers and accumulate in tissues like the liver, placenta, and breast milk [6], although their precise distribution in human organs remains unclear.

The core pathophysiology of T2DM involves insulin resistance and pancreatic β-cell failure, closely intertwined with immune system dysregulation [7]. Impaired insulin signaling activates immune cells, promoting chronic low-grade inflammation and creating a vicious cycle that exacerbates insulin resistance and β-cell damage [8]. Recently, abnormal immune activation, particularly macrophage-mediated chronic low-grade inflammation, has been recognized as a central link connecting environmental factors to IR. The gut, as the primary interface for MPs exposure, and its dynamic interaction with the liver via the “gut–liver axis” serve as the origin for MPs to induce systemic inflammation.

Beyond the well-characterized peripheral pathologies in the liver, adipose tissue, and pancreas, the brain’s role as the master orchestrator of energy metabolism warrants critical attention in the context of microplastic-induced metabolic dysregulation. The hypothalamus, in particular, serves as the central hub for integrating signals that regulate appetite, energy expenditure, and peripheral insulin sensitivity. This region is exquisitely vulnerable to inflammatory insults. MPs are hypothesized to access the central nervous system either by penetrating the blood–brain barrier (BBB) or indirectly via the gut–brain axis [9]. Once present in the Central Nervous System (CNS), they can activate the brain’s indigenous immune sentinels, the microglia. This activation triggers a state of neuroinflammation, which directly impairs hypothalamic insulin signal transduction and compromises the central regulation of systemic glucose homeostasis [10].

**Figure 1:**
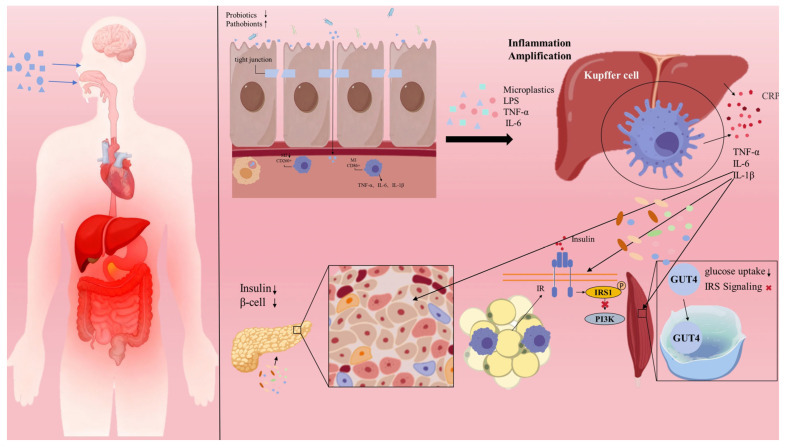
Schematic diagram of the mechanism by which microplastics promote diabetes via the gut–liver axis and macrophage polarization.

Experimental evidence demonstrates that MPs can compromise the intestinal barrier and induce dysbiosis in vivo, allowing harmful bacteria and their metabolites to translocate via the portal vein to the liver. There, they can activate hepatic macrophages (Kupffer cells) and amplify systemic inflammation as shown in animal models [11]. Concurrently, in vitro studies indicate that MPs can induce macrophage M1 polarization via signaling pathways such as TLR4/NF-κB, which is hypothesized to trigger local inflammation and dysfunction in metabolic organs like adipose tissue and pancreas [12]. This review synthesizes the core pathophysiological pathways through which MP exposure precipitates T2DM, with a central focus on the intestinal-liver axis, as illustrated in Figure 1. Once ingested or inhaled, MPs can compromise gut barrier function. They contribute to a “leaky gut” phenotype through three main mechanisms: inducing oxidative stress, disrupting tight junction proteins, and altering the gut microbial ecosystem. Within this compromised environment, intestinal macrophages are activated—both through direct phagocytosis of MPs and indirectly by dysbiotic microbiota and their products—and undergo pro-inflammatory M1 polarization. These activated cells release a cascade of cytokines, including TNF-α, IL-6, and IL-1β, which further exacerbate local inflammation and barrier disruption.

Subsequently, these inflammatory signals and translocated MP particles traverse the portal vein to the liver, where they activate Kupffer cells, again promoting M1 polarization and metabolic reprogramming. These activated hepatic macrophages serve a dual role: first, as local effectors, they secrete inflammatory cytokines that directly impair insulin signaling in hepatocytes, thereby inducing hepatic IR. Second, and more critically, they function as a systemic “inflammatory amplifier,” releasing copious quantities of acute-phase proteins (e.g., C-reactive protein, CRP) and inflammatory mediators into the circulation. This process elevates local inflammation to a state of systemic low-grade inflammation.

This systemic inflammatory milieu then acts synergistically to disrupt the function of peripheral metabolic organs. In the pancreas, the inflammatory environment compromises β-cell function and suppresses insulin secretion. Within adipose tissue, it promotes macrophage infiltration, inciting local inflammation that exacerbates IR and disrupts lipid metabolism. In skeletal muscle, it inhibits insulin-stimulated glucose uptake. Ultimately, the convergence of multi-organ IR and progressive β-cell dysfunction culminates in the pathogenesis of T2DM.

This review systematically consolidates the emerging evidence delineating the role of the intestinal-liver axis in mediating MP-induced metabolic disease. We critically examine the current state of knowledge on how MPs govern macrophage polarization to instigate multi-organ IR and β-cell failure. By highlighting pivotal research controversies and charting future investigative directions, this synthesis offers a novel paradigm for understanding environmental contributions to metabolic disorders.

### Literature Search Strategy and Methods

This narrative review systematically synthesizes the current body of evidence investigating the role of microplastics (MPs) in the pathogenesis of type 2 diabetes mellitus (T2DM), with a specific focus on the gut–liver axis and macrophage-mediated immune-metabolic regulation. To ensure comprehensive and representative coverage of the literature, the following search strategy was employed.

1. Databases Searched: A systematic literature search was conducted across the following electronic databases, covering publications from their inception to October 2025: PubMed/MEDLINE/Web of Science Core Collection/Scopus.
2. Search Strategy and Keywords: A combination of Medical Subject Headings (MeSH) terms and free-text keywords was applied using Boolean operators. The core search string was structured as follows: (microplastics OR nanoplastics OR polystyrene OR polyethylene) AND (diabetes mellitus OR insulin resistance OR glucose metabolism OR metabolic syndrome) AND (gut–liver axis OR intestinal barrier OR gut microbiota) AND (macrophage OR Kupffer cells OR inflammation). Additionally, the reference lists of all included articles were manually screened (using the “snowball” method) to identify any potentially relevant studies missed during the initial database search.
3. Inclusion and Exclusion Criteria: Inclusion Criteria: Studies were considered eligible if they: (1) involved exposure to microplastics or nanoplastics; (2) addressed metabolic phenotypes such as diabetes, insulin resistance, glucose dysregulation, or obesity; (3) investigated mechanisms related to the intestine, liver, macrophages, or inflammatory pathways; (4) were original research articles (in vivo or in vitro), comprehensive reviews, systematic reviews, or prospective cohort studies; (5) were published in English.
4. Exclusion Criteria: Studies were excluded if they: (1) focused exclusively on the environmental fate, detection, or characterization of microplastics without investigating biological or toxicological effects; (2) were conference abstracts, commentaries, opinion pieces, letters to the editor, or patents; (3) lacked full-text availability or contained critically insufficient data.
5. Literature Quality Assessment: Given the substantial heterogeneity in study design—encompassing animal models, in vitro cell systems, and human observational studies—a unified quantitative scoring system for meta-analysis was not employed. However, to ensure the robustness of the synthesized evidence, priority was given to studies published in reputable peer-reviewed journals that demonstrated rigorous experimental design (e.g., inclusion of appropriate control groups, adequate sample sizes, and sound statistical methodologies). For human studies, preference was given to prospective cohort or well-designed case–control studies that adjusted for key confounding factors. Contentious or contradictory findings are critically discussed within the main text to provide a balanced perspective.

## 2. Overview of Microplastics

Plastics are synthetic or semi-synthetic materials composed of polymers such as polypropylene (PP), polystyrene (PS), polyethylene terephthalate (PET), polyvinyl chloride (PVC), polyethylene (PE), and polyurethane (PUR), often combined with chemical additives to form diverse products [12,13].

The continuous growth in plastic production, coupled with inadequate waste management, has led to massive environmental plastic pollution, posing a persistent and potentially irreversible ecological threat [14]. Environmental plastics fragment via abiotic processes (e.g., photo-degradation, thermal oxidation) and biodegradation into particles of varying sizes: megaplastics (>1 m), macroplastics (1 m–2.5 cm), mesoplastics (2.5 cm–5 mm), microplastics (5 mm–1 μm), and further into nanoplastics (<0.1 μm) [15]. MPs are persistent and mobile, prevalent not only in marine environments (over 70% of marine debris contains plastic), surface water, and groundwater, but also transported long distances via wind and precipitation. Indoor air MPs concentrations are significantly higher than outdoors [16,17]. Once fragmented into MPs or NPs, their environmental hazard increases. Smaller MPs can adsorb heavy metals and microorganisms, facilitating co-contaminant dispersal [18]. MPs enter humans via the marine food chain and directly from food in plastic packaging, indicating unavoidable human exposure [19]. Studies show MPs < 10 μm can cross cell membranes and have been detected in placental tissue [4]. Thus, as MPs degrade into smaller NPs, the risk of cellular and tissue membrane disruption escalates [20].

### Metabolic Toxicity of Microplastics

The pervasive environmental contamination by MPs has precipitated a pressing scientific inquiry into their potential impact on human health. Chronic accumulation and exposure to MPs are implicated in the pathogenesis of multisystem toxicity, driving histopathological aberrations and functional deficits within the digestive, immune, reproductive, and endocrine systems [21]. Emerging evidence positions MPs as a potential contributory factor in the etiopathogenesis of metabolic disorders, notably diabetes mellitus. The proposed mechanisms are multifaceted, encompassing the induction of oxidative stress, systemic inflammation, and endocrine disruption. These perturbations collectively impair insulin signal transduction, promote hepatic steatosis, induce gut microbiota dysbiosis, and compromise adipose tissue functionality, thereby fostering IR and the progression of diabetes [22,23]. At a molecular level, MP accumulation in key metabolic organs such as the liver and kidneys has been shown to suppress critical signaling cascades, including the IRS1/PI3K/AKT pathway, while concurrently promoting the release of pro-inflammatory cytokines. The implications may extend transgenerationally, with studies suggesting that gestational MP exposure could be a risk factor for gestational diabetes, placental dysfunction, and adverse pregnancy outcomes [24].

Despite these concerning findings from preclinical models, a critical translational gap persists. Direct, unequivocal evidence establishing a causal link between MP exposure and specific human diseases remains elusive [21]. This evidentiary chasm is likely attributable to the complexity of human metabolic physiology and the characteristically low concentrations of MPs in real-world scenarios. Indeed, recent investigations suggest that the cytotoxic effects observed in mammalian cells may be less catastrophic than initially hypothesized, often manifesting only at concentrations substantially exceeding typical environmental levels [25]. The observation that over 90% of ingested MPs are rapidly eliminated via fecal excretion further supports the argument for minimal acute human health impacts. However, this perspective warrants critical scrutiny. The insidious nature of MP pollution lies not in acute high-dose events, but in its defining characteristics: chronic, low-dose exposure. This paradigm necessitates a focus on bioaccumulation, wherein even a highly efficient excretory system permits the gradual, lifelong accrual of particles, potentially leading to a growing subpopulation of cells—particularly macrophages—with high internalized MP burdens [26].

At environmentally relevant concentrations, MP-induced cytotoxicity is often markedly lower than effects observed in high-dose laboratory studies. Multiple studies demonstrate that when exposure levels approximate human real-world scenarios, toxic effects are significantly attenuated or undetectable. For instance, PS-MPs showed no significant impact on the viability or apoptosis of human colonic and small intestinal epithelial cells, with only minor changes in oxidative stress and mitochondrial membrane potential observed at the highest concentrations. Similarly, long-term exposure to PP-MPs revealed that initial effects diminished over time, suggesting potential adaptive or compensatory mechanisms within biological systems [27].

The cytotoxic potential of MPs exhibits a pronounced dependence on both particle size and cell type. Smaller particles are more readily internalized by phagocytes such as macrophages, triggering a more robust toxic response. Conversely, while larger particles are engulfed less frequently, their sheer volume can still perturb cellular function by physically occupying intracellular space [28]. Particle morphology is equally critical; irregularly shaped MPs are more potent inducers of cell death compared to their spherical counterparts. Human colonic adenocarcinoma cells, in particular, demonstrate heightened sensitivity. Quantitative studies have begun to define toxicity thresholds at environmentally relevant concentrations: concentrations as low as 10 μg/mL (for 5–200 μm particles) can impair cell viability, while 20 μg/mL (for 0.4 μm particles) is sufficient to induce cytokine release. These findings underscore that while acute toxicity may be low, chronic, low-grade exposure still poses a potential threat to specific, vulnerable cell populations.

The heterogeneity of findings across the literature is a product of numerous variables, including the physicochemical properties of the MPs (polymer type, size, shape, surface functionalization), exposure paradigms (dose, duration), and the physiological state of the exposed organism. A critical variable is host health status; for example, diabetic models exhibit heightened metabolic sensitivity to MP insult compared to healthy controls, where effects may be largely confined to gut microbiota-mediated inflammation [29]. Furthermore, the preponderance of studies employing acute or subacute exposure protocols limits their extrapolation to the human condition of lifelong, low-level exposure. Intriguingly, some long-term studies suggest that initial adverse effects may wane over time, hinting at the presence of homeostatic or adaptive responses.

A fundamental methodological constraint pervades the field: the exposure doses used in most animal and cellular studies vastly exceed human real-world exposure levels. Human contact with MPs occurs primarily through ingestion and inhalation. Estimates place the average daily intake at 0.2–1.2 × 10^6^ particles from drinking water, 4.9–5.8 × 10^5^ particles from food, and 0.2–2.5 × 10^6^ particles via inhalation [4,5]. In mass terms, MP concentrations in drinking water typically fall within the range of 10^−3^–10^1^ μg/L, and in food, approximately 10^−2^–10^2^ μg/kg wet weight [30]. In stark contrast, rodent studies frequently employ oral gavage doses of 0.1–100 mg/kg/day, which, following allometric scaling, equate to human equivalent doses up to four orders of magnitude higher. Similarly, in vitro studies often use concentrations of 50–1000 μg/mL, levels far exceeding any plausible human physiological exposure [31,32].

However, dismissing the biological risk of MPs based solely on the numerical disparity between environmental and experimental doses may lead to a critical underestimation of their true pathogenic potential. The paradigm of chronic accumulation is paramount. While the majority of ingested mass is excreted, a fraction of non-degradable particles persists and accumulates in tissues. A lifetime exposure model estimates a cumulative body burden of up to 5.01 × 10^4^ particles in a 70-year-old adult [33]. This accumulation is not uniform; it leads to the formation of localized, high-concentration microenvironments within specific tissue niches, such as the Peyer’s patches of the intestine or Kupffer cells in the liver. This phenomenon of tissue-specific internal dose is critical. Macrophages, for instance, exhibit a remarkable capacity for internalizing NPs—with efficiencies far exceeding ambient concentrations—yet are unable to degrade them. This cellular-level enrichment means that specific immune cells can be subjected to a high particulate load even against a backdrop of low systemic exposure, creating a “hotspot” for potential cytotoxicity [26,31]. The dose–response relationship is not strictly linear. As noted, thresholds for effects like reduced cell viability (10 μg/mL) or cytokine release (20 μg/mL) exist at relatively low concentrations [27]. Furthermore, the host’s physiological state acts as a powerful effect modifier. The heightened sensitivity of diabetic animal models demonstrates that a pre-existing disease state can lower the threshold for MP toxicity, effectively transforming a previously innocuous dose into a pathogenic cofactor [23].

In conclusion, the pathogenic effects of MPs are conditional, manifesting most clearly under a specific convergence of factors: chronic exposure, small particle size, irregular morphology, vulnerable cell types, or a compromised host physiological state. Therefore, a nuanced risk assessment framework is imperative—one that moves beyond simple dose extrapolation to integrate the complex interplay of particle characteristics, individual susceptibility, and the dynamics of chronic bioaccumulation. While MPs constitute a widespread, recalcitrant environmental pollutant with the potential to contribute to metabolic and other systemic diseases, their most significant threat to public health may lie not in overt toxicity, but in the subtle and hitherto overlooked biological mechanisms they initiate. The following sections will systematically delineate how ingested MPs, acting primarily through the gut, can exploit the gut–liver axis to progressively destabilize systemic glucolipid metabolic homeostasis.

## 3. Impact of Microplastics on the Intestine

MPs are ubiquitous in surface water, groundwater, and wastewater. Annual maximum intake for adults is estimated at 458,000 particles from tap water and 3,569,000 particles from bottled water [30]. Studies across species confirm ingested MPs accumulate in the gut. In rats, approximately 10% of orally administered 60 nm PS-NPs were found in the gastrointestinal tract after 5 days [34]. While MPs > 150 μm are not absorbed, they bind to the intestinal mucus layer and directly contact the apical surface of epithelial cells, potentially causing local inflammation and immune effects. Multiple uptake mechanisms exist, including enterocyte endocytosis, translocation via M-cells, adsorption, and paracellular uptake. In rodents, particles are taken up via endocytosis, transcellular, and paracellular routes without overt barrier disruption [35]. One study found only 0.3% of orally administered latex particles crossed the epithelium. Despite low levels, intestinal particle absorption can lead to systemic exposure. Absorbed NPs distribute to the liver, spleen, heart, and even brain, potentially exerting systemic toxicity.

### 3.1. Microplastics Disrupt the Intestinal Epithelial Barrier

MPs-induced intestinal barrier damage is species-general, with core mechanisms involving oxidative stress, downregulation of tight junction proteins, and reduced mucus secretion. Caenorhabditis elegans exposed to mixed MPs (PS/PP/PE/PVC, 0.1–70 μm) for 2 days showed reduced intestinal calcium levels and increased Glutathione S-transferase (GST) expression [36]. Mussels exposed to PS, PP, or PET MPs (2 mg/L) alone or combined with Cd (30 μg/L) for 7 days exhibited decreased catalase (CAT) and glutathione reductase (GR) antioxidant enzyme activities in the mantle, increased lipid peroxidation, and reduced digestive enzyme activities in the digestive gland [37]. Zebrafish exposed to 50 nm PS-NPs (0.07 mg/L) for 21 days showed significant downregulation of cat and upregulation of gpx1a and sod1 in the liver, indicating activated oxidative stress. Electron-dense particles resembling NPs accumulated near intestinal microvilli, suggesting gut retention and potential impact on structure and function [38].

Evidence of MPs intestinal toxicity in mammals is emerging. Mice exposed to PS-MPs (0.5 μm, 50 μm; 100 μg/L, 1000 μg/L) for 5 weeks showed reduced colonic mucus secretion and significantly decreased Muc1 gene transcription (a highly glycosylated transmembrane mucin with protective functions) [39]. PE-MPs (10–150 μm; 2, 20, 200 μg/g) for 5 weeks induced colon and duodenum inflammation and upregulated TLR4, AP-1 (c-Jun), and IRF5 protein levels [40]. PE-MPs (45–53 μm; 100 mg/kg/day) for 30 days impaired intestinal permeability and altered gene expression (downregulated Cyp1a2, Cyp1a5; upregulated Rdh16, Gm8909) [41]. Mice gavaged with PS-MPs (1 mg/kg/day; 0.2, 1, 5 μm) for 28 days developed colonic oxidative stress, inflammatory cell infiltration, elevated pro-inflammatory cytokines, increased permeability, reduced mucus, and downregulated tight junction proteins (ZO-1, OCLN, CLDN-1), with 5 μm PS-MPs causing the most damage. In vitro Caco-2 experiments confirmed 5 μm PS-MPs activate the NF-κB/NLRP3/MLCK pathway via Reactive Oxygen Species (ROS) induction [42]. Mdr2 mice exposed to 0.5 μm PS-MPs (200 μg/day) showed exacerbated liver collagen deposition, inflammation, elevated serum Alkaline phosphatase (ALP)/Gamma-Glutamyl Transferase (γ-GGT), reduced colonic goblet cells, and downregulated intestinal barrier proteins (ZO-1, Occludin, Claudin-1) [43].

In summary, MPs impair intestinal barrier function via oxidative stress, tight junction protein downregulation, and reduced mucus secretion, increasing permeability—a common toxic mechanism. Barrier integrity is paramount for gut homeostasis. Its compromise by MPs not only facilitates translocation of harmful luminal contents but also inevitably disrupts the ecological balance of the gut microbiota.

### 3.2. Microplastics Induce Gut Dysbiosis

MPs exposure significantly disrupts gut microbiota homeostasis in a dose- and duration-dependent manner. In mice, long-term exposure to PS-MPs (5 μm, 1000 μg/L, 5–6 weeks) caused substantial phylum-level compositional changes [39,44]. Effects vary between studies: both reported decreased Alphaproteobacteria abundance; Lu et al. found reduced Actinobacteria and Firmicutes, while Luo et al. observed increased Actinobacteria with no significant change in Proteobacteria or Firmicutes. A key difference was Luo’s exposure during gestation and lactation [45].

An adult mucosal artificial colon model combined with intestinal epithelial and mucus-secreting cell co-cultures analyzed repeated PE-MPs exposure effects on human gut microbiota and barrier. PE-MPs impacts were donor-dependent, with characteristic core microbiota shifts: increased abundance of potentially harmful pathobionts (Desulfovibrionaceae, Enterobacteriaceae) and decreased beneficial taxa (Christensenellaceae, Akkermansiaceae), accompanied by altered volatile organic compound profiles [46]. PE-MPs (10–150 μm) in mouse feed (2, 20, 200 μg/g) for 5 weeks increased Firmicutes, Deferribacteres, and Staphylococcus, while decreasing Bacteroidetes and Parabacteroides [40]. PE-MPs (45–53 μm; 100 mg/kg/day via gavage for 30 days) increased *Actinobacteria*, *Lactobacillus*, *Adlercreutzia*, *Butyricimonas*, and *Parabacteroides* [41]. A machine learning meta-analysis of 1352 gut microbiota samples from six animal species indicated mice are significantly more sensitive to MPs toxicity than other animals, showing reduced diversity, increased Firmicutes/Bacteroidetes ratio, disrupted microbial networks, and beneficial/harmful bacteria imbalance. Unlike earthworms and insects, mice have limited MPs degradation capacity, with exposure duration being a core driver of microbiota changes [8].

In conclusion, all animal studies on MPs exposure report gut dysbiosis. Although specific changes vary due to MPs type, dose, and model, multiple lines of evidence converge that MPs exposure significantly disrupts gut microbiota stability, reducing diversity, altering composition, and shifting the balance between beneficial and pathogenic bacteria. Given the intimate crosstalk between gut microbiota and host immunity, MPs-induced dysbiosis often coincides with increased pathobionts and decreased beneficial bacteria, potentially disrupting local immune balance and sending persistent activation signals to gut-resident macrophages.

### 3.3. MPs-Induced Macrophage M1 Polarization and Intestinal Barrier Damage

Macrophages are ubiquitous in human tissues, with tissue-specific names: alveolar macrophages (lungs), microglia (CNS), osteoclasts (bone), and Kupffer cells (liver). They are central to tissue development, repair, homeostasis, pathogen defense, chronic inflammation regulation, fibrosis, and cancer suppression [47]. The intestinal, pulmonary, and dermal barriers are primary MPs entry routes, where macrophages play crucial roles. As the predominant phagocytes in the mucosal microenvironment and the largest cell population in innate immunity, macrophages are indispensable for recognizing and clearing pathogenic endogenous and exogenous substances across tissues [48]. They defend against microbes and toxins and can act as carriers, transporting MPs particles systemically [49]. Dysfunction of these “frontline” immune cells can thus indirectly compromise barrier integrity. In MPs-induced gut barrier damage, macrophages serve as a hub connecting “physical barrier disruption”, “biological barrier dysbiosis” and “immune barrier activation”.

#### 3.3.1. Phagocytosis and Accumulation of Microplastics

Upon entry, MPs first activate host defenses like mucus secretion and immune cell recruitment, particularly macrophages. Studies on nanoplastic distribution and bioaccumulation show significant enrichment within macrophages. Experiments with 1–4 μm MPs demonstrated murine macrophages can internalize MPs particles [50].

Macrophages internalize but do not degrade MPs [51]. The internalization behavior and toxicity of MPs in macrophages exhibit significant size dependence: small (0.5 μm) PS-MPs are phagocytosed more efficiently than larger (5 μm) particles and more readily induce oxidative stress, mitochondrial dysfunction, and apoptosis. Furthermore, PS-MPs drive macrophages toward a pro-inflammatory M1 phenotype, upregulating associated markers and inflammatory cytokines [31]. Additional studies indicate that 0.5 μm MPs exert stronger toxicity than 5 μm MPs due to their greater cellular uptake and significant promotion of ROS generation, which triggers oxidative stress and metabolic reprogramming. MPs exposure upregulates pro-inflammatory cytokines (TNF-α, IL-1β) and pro-apoptotic genes (*Caspase 3*, *Caspase 7*, *Caspase 9*, *Bax*), while downregulating the anti-apoptotic gene Bcl-2 and key sphingolipid metabolites such as sphingosine. These changes ultimately drive macrophage apoptosis through both mitochondrial-dependent and independent pathways [32]. Together, these findings demonstrate that MPs size is a critical determinant of their internalization by macrophages and the subsequent immunotoxic responses. Although MPs may be passively released upon macrophage death, viable macrophages lack active excretion mechanisms. Thus, internalized particles persist during the cell’s lifetime, potentially leading to intracellular accumulation [52]. MPs can also induce lysosomal damage, triggering macrophage reprogramming and ultimately upregulating IL-1β expression [53]. While these observations confirm that macrophages uptake MPs and that MPs exert cytotoxic effects, they further suggest that MPs toxicity is closely associated with their intracellular accumulation.

#### 3.3.2. Dysbiosis and PRR Signaling Pathways

Multiple studies show chronic MPs exposure in mice causes dysbiosis, impaired barrier function, and metabolic disorders. Environmental MPs often enrich opportunistic pathogens and reduce beneficial taxa. The relationship between probiotics and barrier integrity suggests dysbiosis plays an important indirect role in MPs-induced gut dysfunction. Dysbiosis can lead to abnormal pattern recognition receptor (PRR) expression and signaling, prompting macrophages to produce pro-inflammatory cytokines like IL-6 and TNF-α, aggravating intestinal inflammation [54].

Peptidoglycan recognition proteins (PGLYRPs) are key in gut microbiota regulation. PGLYRP1 is an essential receptor for Lysine N-acetylglucosamine N-acetylmuramic tripeptide (GMTriP-K) activation of innate immunity. In intestinal macrophages, PGLYRP1 forms a complex with Nucleotide-binding oligomerization domain 2 (NOD2) and GEF-H1, collectively regulating GMTriP-K-induced gene expression. Upon GMTriP-K stimulation, endoplasmic reticulum-localized PGLYRP1 interacts with NOD2 in the Golgi, initiating signal cascades [55]. Dysbiosis activates gut macrophages via various PRRs, triggering inflammation and barrier damage. NOD2, TLRs, Mincle, and other PRRs are crucial in macrophage function, gut immune homeostasis, and disease. Although direct evidence of MPs activation of PRRs is limited, MPs can indirectly induce PRR-mediated macrophage activation and inflammation by altering gut microbiota structure. Dysbiosis abnormally activates PRR signaling, leading to barrier impairment and chronic inflammation. Studies show PS-MPs disrupt the gut microbiome, impair barrier function, exacerbate peripheral inflammation, and alter serum metabolomes. Moreover, dysbiosis-derived LPS can activate macrophage TLR4, inducing hypoxia inducible factor 1 subunit alpha (HIF-1α) expression, which initiates aerobic glycolysis, enhancing ATP production and promoting a pro-inflammatory macrophage phenotype [56].

#### 3.3.3. M1 Polarization and the “Leaky Gut” Vicious Cycle

Studies show ingested MPs form a specific protein corona in the harsh gastrointestinal environment, which may enhance phagocytosis by THP-1-derived human macrophages [57]. Adult zebrafish exposed to 500 μg/L PS-M/NPs (100 nm, 5 μm, 200 μm) showed increased intestinal M1 and decreased M2 macrophage proportions [58]. In RAW264.7 macrophages, 50 μg/mL PS-NPs (50 nm and 500 nm) significantly increased CD86, iNOS, and TNF-α expression and decreased CD206, IL-10, and Arg-1 expression, indicating M1 polarization [59]. LPS-stimulated macrophages exhibit enhanced glycolysis, inhibited Succinate dehydrogenase (SDH) activity, and increased IL-1β and TNF-α secretion. Notably, ameliorating PS-MPs-induced dysbiosis significantly reduced serum LPS, IL-1β, IL-6, and TNF-α levels, helping prevent gut barrier damage and peripheral inflammation, and inhibited the TLR4/MyD88/NF-κB pathway, mitigating neuroinflammation [60].

Co-culture of differentiated human intestinal Caco-2 epithelial cells with HT29-MTX-E12 mucus-secreting goblet cells, closely coupled with differentiated THP-1 macrophage-like cells or primary human monocyte-derived macrophages, showed significant “leaky gut” features upon inflammatory stimulation: severe impairment of epithelial integrity with reduced Trans-Endothelial/Trans-Epithelial Electrical Resistance (TEER), decreased tight junction protein expression, increased FITC-dextran permeability, and massive release of TNF-α and IL-6. Interestingly, IL-23 release was not detected in M1-like THP-1 co-cultures but was present with primary human M1 macrophages [61].

IL-6 upregulates Caudal-type homeobox transcription factor 2 (Cdx2) via MEK/ERK and PI3K pathways, inducing claudin-2 expression and increasing tight junction permeability [62]. TNFα (1000 U/mL) incubation of a jejunal epithelial cell model significantly reduced TEER, increased [^3^H]-D-mannitol permeability, and downregulated occludin, claudin-1, and claudin-3 expression [63]. Recent findings highlight IL-1β-induced increased gut permeability as a key contributor to intestinal inflammation via NF-κB activation, Myosin light-chain kinase (MLCK) gene activation, and microRNA-mediated post-transcriptional regulation of occludin [64].

Barrier damage allows luminal antigens and bacteria to infiltrate the lamina propria, activating immune cells and producing more inflammatory cytokines. Sustained inflammation leads to further TNF-α, IL-1β, and IL-6 secretion, exacerbating barrier damage—a vicious cycle of “leaky gut” [65].

In summary, MPs either directly phagocytosed by macrophages or indirectly via dysbiosis activate macrophages, triggering NF-κB signaling and metabolic reprogramming, driving M1 polarization and massive release of TNF-α, IL-1β, and IL-6. This aggravates intestinal inflammation and barrier damage, initiating a vicious cycle of “barrier disruption-antigen penetration-immune activation-increased permeability.” This local inflammatory sentinel and compromised barrier further opens a channel for MPs and bacterial products to enter the portal vein, reaching the liver—the central metabolic-immune organ—thereby expanding local inflammation into a systemic state.

## 4. Hepatic and Systemic Inflammation

Following gut barrier disruption, MPs and associated harmful substances enter the liver via the portal vein, activating hepatic immune cells, particularly resident Kupffer cells and infiltrating macrophages. The liver is not only a key site for MPs metabolism but also acts as an “inflammation amplifier”, escalating local gut-derived signals into a systemic low-grade inflammatory state, leading to multi-organ dysfunction and systemic metabolic dysregulation.

### 4.1. Activation of Hepatic Macrophages and Liver Inflammation

The liver is among the first organs to process harmful substances absorbed from the gut and plays a pivotal role in MPs metabolism. Ingested MPs that evade digestive clearance enter the liver directly via the portal vein, accumulate, and induce pathological changes leading to injury. MPs exposure in mice induces hepatic metabolite reprogramming (downregulation of allantoin, polydatin; upregulation of D-glutamine, ergosterol, 1,3,5-trihydroxybenzene) and antioxidant defense imbalance (ROS accumulation, decreased CAT, Superoxide dismutase (SOD), Glutathione peroxidase (GSH-Px) with compensatory later increases), creating an oxidative stress microenvironment that mediates liver inflammation [66]. PS-MPs clearly induce hepatic inflammation: mice orally exposed to 5 μm MPs for 28 days showed elevated serum LPS, TNF-α, IL-6, IL-1β, and liver injury markers ALT/AST, indicating systemic and local inflammation activation via the TLR4/MyD88/NF-κB pathway, upregulating hepatic inflammatory genes and proteins [67].

Macrophages are the primary immune cells in the liver, including resident Kupffer cells and bone marrow-derived infiltrating macrophages. These hepatic macrophages are highly heterogeneous and crucial in liver disease, making them potential targets influencing fibrosis progression and resolution. MPs increase hepatic macrophage infiltration and induce polarization towards a pro-inflammatory M1 phenotype (upregulated M1, downregulated M2) [68]. MPs promote macrophage infiltration, polarization, metabolic reprogramming, and macrophage extracellular trap (MET) formation, exacerbating inflammation and fibrosis via ROS, NF-κB/NLRP3, and TLR4 signaling [51,69,70]. MPs increase inflammatory cytokines (IL-6, IL-8, TNF-α) and promote cell death in advanced human liver models, with Kupffer cells being the primary target for accumulation and toxicity [71]. Furthermore, chronic PS-MPs exposure promotes pyroptosis in hepatic macrophages, significantly aggravating acute liver injury [72]. Orally ingested MPs phagocytosed by Kupffer cells induce M1 polarization, activating NF-κB signaling and releasing pro-inflammatory cytokines (TNF-α, IL-1β, IL-6) while suppressing anti-inflammatory factors. MPs also promote NK cell infiltration and maturation and reduce immunosuppressive B cells, collectively exacerbating hepatic inflammation and tissue damage [68].

### 4.2. Hepatic Macrophages Induce Hepatic Insulin Resistance

As the central metabolic organ, liver inflammation is increasingly linked to IR. Hepatic macrophages, core components of the liver immune system, are closely associated with inflammation and IR development. Inflammatory hepatic macrophages shift from M2 to M1, secreting TNF-α, IL-6, etc., directly interfering with insulin signaling, increasing hepatic gluconeogenesis, and impairing glucose uptake.

Activation of the vitamin D receptor (VDR) in hepatic macrophages significantly suppresses inflammation, ameliorates steatosis, and improves insulin sensitivity in diet-induced obese mice. VDR agonist calcipotriol reduced hepatic inflammation and fat accumulation, improving insulin sensitivity specifically in the liver, not muscle or fat. Depleting hepatic macrophages abolished these benefits, confirming VDR activation in macrophages is key to improving IR [73]. Selective depletion of Kupffer cells completely prevented high-fat or high-sucrose diet-induced hepatic steatosis and IR in mice. In vitro, M1-polarized Kupffer cells secreted TNF, inhibiting hepatocyte fatty acid oxidation, promoting triglyceride accumulation, and reducing insulin sensitivity—effects reversed by TNF-neutralizing antibody, identifying TNF as the core mediator [74]. Using Induced Pluripotent Stem Cells-derived hepatocytes (iPSC-Heps) with reconstructed insulin signaling and glucose metabolism, co-culture with pro-inflammatory iPSC-derived macrophages induced significant hepatocyte IR—impaired insulin suppression of gluconeogenesis/glycogenolysis and aberrant glycolysis—even without lipid deposition. Mechanistic screening identified TNFα and IL-1β as core effectors; dual neutralization more effectively restored insulin sensitivity than single blockade, operating via NF-κB and JNK pathways [75]. In High-Fat Diet (HFD)-induced obese mice, hepatic macrophages infiltrated and M1-polarized. Activated M1 bone marrow-derived macrophages secreted exosomes enriched with miR-143-5p, which, upon hepatocyte uptake, targeted and inhibited dual-specificity phosphatase MKP5 (Dusp10). MKP5 downregulation reduced Akt and GSK-3β phosphorylation, impairing glycogen synthesis and inducing hepatocyte IR [76]. Contrary to the classic inflammatory cytokine model, liver macrophages in fly, mouse, and human obesity-associated IR did not switch to a typical pro-inflammatory phenotype. Instead, they secreted non-inflammatory factors like insulin-like growth factor-binding protein 7 (IGFBP7), which bound insulin receptor, activated ERK signaling, promoted lipogenesis and gluconeogenesis, and induced IR. Notably, IGFBP7 mRNA in obese IR patients showed increased RNA editing, potentially generating a variant with stronger insulin receptor binding affinity [77]. In obese IR individuals, hepatic NRF2 expression is downregulated, impairing the response to lipid accumulation. This NRF2 dysfunction correlates with upregulated miR-144 in hepatic macrophages, which directly targets NRF2. Silencing miR-144 in hepatic macrophages restored NRF2 and its antioxidant response, improving oxidative stress and insulin signaling [78]. In summary, hepatic macrophages induce IR not only via classic inflammatory pathways (TNF-α/IL-1β) but also through non-inflammatory factors (IGFBP7), exosomes, and oxidative stress, highlighting their potential as core intervention targets.

Hepatic macrophages induce IR via dual mechanisms: inflammatory pathway interference and metabolic reprogramming. M1 macrophages secrete TNF-α and IL-1β, which disrupt insulin signaling: IKKβ and JNK kinases catalyze IRS1 serine phosphorylation, inhibiting its tyrosine phosphorylation and promoting degradation, while also inhibiting Akt activation [75,79]. Selective Kupffer cell depletion completely prevents HFD-induced steatosis and IR; TNF neutralization reverses M1 macrophage-mediated suppression of hepatocyte insulin sensitivity [75].

Simultaneously, MPs phagocytosed by hepatic macrophages induce metabolic reprogramming: a shift from oxidative phosphorylation to glycolysis-dominated activation, characterized by enhanced glycolysis, reduced mitochondrial respiration, and lactate/succinate accumulation [51]. Lactate interferes with hepatocyte insulin response; succinate stabilizes HIF-1α, promoting IL-1β expression, forming a glycolysis-inflammation cycle. Macrophages also compete with hepatocytes for glucose, further impairing insulin-mediated metabolism [32]. Additionally, hepatic macrophages can induce IR via non-inflammatory mechanisms, e.g., secreting IGFBP7 to bind insulin receptor or via miR-144 suppressing NRF2, exacerbating oxidative stress and metabolic dysregulation [77,78].

In summary, animal and cellular studies consistently show MPs phagocytosis by hepatic macrophages disrupts their function, induces inflammation and oxidative stress, significantly perturbs hepatic lipid and glucose homeostasis, and increases metabolic disease risk. Hepatic macrophages under MPs exposure promote hepatic and systemic IR via direct interference with inflammatory pathways (IRS1/Akt) and indirect production of harmful metabolites via metabolic reprogramming, underscoring the centrality of immunometabolic crosstalk in environmentally induced metabolic disease. The long-term health impacts of chronic exposure warrant high concern.

### 4.3. The Liver as a Systemic Inflammation Amplifier

MPs breaching the gut barrier enter the portal system, reach the liver, activate macrophages, and trigger inflammatory responses and acute-phase protein synthesis. As the largest metabolic and immunoregulatory organ, the liver transforms local inflammatory signals into a systemic low-grade inflammatory state, releasing cytokines and acute-phase proteins into systemic circulation via the hepatic vein, affecting distant organs. This involves not only direct cytokine release but also synthesis and secretion of liver-specific acute-phase proteins, forming a complex amplification network that establishes chronic low-grade inflammation, increasing risk for various diseases.

The liver, as the metabolic hub, directly participates in and amplifies systemic low-grade inflammation by secreting cytokines and acute-phase proteins. Liver-synthesized positive acute-phase proteins (e.g., C-reactive protein CRP, fibrinogen, serum amyloid A (SAA)) and negative ones (e.g., hepcidin, albumin) are released into circulation via hepatic sinusoids, serving as sensitive biomarkers. In chronic low-grade inflammation, IL-6 is a key cytokine promoting acute-phase protein synthesis. Elevated IL-6 rapidly induces hepatic synthesis of CRP, fibrinogen, etc., whose plasma levels rise significantly.

The liver is both a synthesis site and an amplification center. IL-1β inhibits production of proteins like γ-fibrinogen and α_2_-macroglobulin while strongly upregulating CRP, SAA, and hepcidin. STAT3-NF-κB signaling interactions allow for precise inflammation regulation; imbalance leads to sustained circulating inflammatory factors [80,81].

Acute-phase proteins are not just markers but actively participate in and amplify inflammation: directly binding pathogens/damaged cells, promoting complement activation and phagocytosis [82]; interacting with immune cells to promote release of IL-6, TNF-α, IL-8, activating NF-κB and amplifying local/systemic inflammation [80]; some form positive feedback loops via specific receptors, perpetuating inflammatory signaling [81].

The portal vein collects blood from digestive organs, directing it to the liver for processing. Hepatic sinusoids are highly permeable, facilitating exchange. Portal blood, after hepatic metabolism, enters systemic circulation via the hepatic vein and inferior vena cava. In portal hypertension, sinusoidal endothelial tight junctions may be disrupted, increasing permeability and accelerating cytokine release. Studies show portal pressure correlates positively with serum CRP and IL-6 [83,84]. MPs can induce chronic hepatic inflammation, activate NF-κB, promote fibrosis, and increase portal pressure [82], suggesting MPs may affect inflammatory factor release efficiency by altering portal pressure.

In summary, hepatic macrophages are central drivers of IR, interfering with glucose and lipid metabolism via multiple avenues. Exogenous MPs phagocytosed by hepatic macrophages induce metabolic reprogramming towards glycolysis and exacerbate oxidative stress, aggravating hepatic inflammation. The liver, as an inflammation amplifier, receives signals via the portal vein, synthesizes abundant acute-phase proteins and cytokines, and releases them into circulation, upgrading local inflammation to a systemic chronic low-grade inflammatory state that elevates metabolic disease risk systemically.

## 5. Multi-Organ Dysfunction Under Systemic Inflammation

MPs instigate gut microbiota dysbiosis and are directly phagocytosed by intestinal resident macrophages, driving their M1 polarization. The ensuing release of pro-inflammatory cytokines such as TNF-α and IL-1β disrupts tight junction proteins, initiating a vicious “leaky gut” cycle. These inflammatory signals and translocated particles enter the liver via the portal vein, activating Kupffer cells, which amplify the inflammatory response by releasing copious acute-phase proteins and pro-inflammatory mediators into the circulation, thereby establishing systemic low-grade inflammation. This systemic inflammation leads to coordinated macrophage activation across multiple metabolic organs. In adipose tissue, macrophages form crown-like structures. In pancreatic islets, M1-polarized macrophages impair β-cell function through exosome-mediated signals. In skeletal muscle, resident macrophages suppress glucose uptake via paracrine IL-6 signaling. Although the activation mechanisms differ subtly among organs—driven by saturated fatty acids in adipose tissue and exosomal signals in the pancreas—the core signaling pathways (NF-κB, TLR4) and effector cytokines (TNF-α, IL-1β) are highly conserved, forming a cross-organ immuno-metabolic network that synergistically promotes IR and β-cell failure. Notably, MPs can also traverse the blood–brain barrier or exploit the gut–brain axis to reach the hypothalamus, where they activate microglia and induce neuroinflammation, further disrupting central insulin signaling and energy homeostasis, thereby exacerbating systemic metabolic dysregulation (Table 1).

### 5.1. Pancreatic β-Cell Damage

The pancreas is central to insulin synthesis and secretion, dependent on a balanced local immune microenvironment. Systemic inflammation triggered by MPs via the gut–liver axis allows circulating pro-inflammatory cytokines (TNF-α, IL-6) and chemokines (e.g., CCL2) to disrupt pancreatic immune barriers, recruiting peripheral monocytes to islets where they differentiate into mature macrophages in the inflammatory milieu [85]; these, together with resident islet macrophages, activate, forming a predominantly pro-inflammatory population.

In obesity-related metabolic disease, systemic inflammatory infiltration activates islet macrophages towards M1 polarization. M1 macrophages release TNF-α, IL-1β, directly impairing β-cell function, and exacerbate insulin secretion dysfunction via exosome-mediated communication. M1 macrophage-derived exosomes are taken up by β-cells, delivering miR-212-5p that targets and inhibits SIRT2, disrupting the Akt/GSK-3β/β-catenin pathway and impairing glucose-stimulated insulin secretion [86].

Under systemic inflammation, hepatocyte-derived lipotoxic small extracellular vesicles mediate inflammation in distant organs. In metabolic dysfunction-associated steatotic liver disease, lipotoxic hepatocyte-derived small extracellular vesicles enter circulation and are preferentially taken up by islet macrophages, activating intracellular TLR4 signaling and triggering NF-κB-mediated inflammation and IL-1β release. This creates a local islet inflammatory environment, downregulating key β-cell genes (*Pdx1*, *Gck*, *Ins1*, *Ins2*), activating ER stress and inflammasome pathways, impairing glucose-stimulated insulin secretion and causing β-cell dysfunction [87]. Recent studies find obesity-associated systemic inflammation infiltrates the pancreas, activating islet macrophages and impairing β-cell function and survival. Macrophage-secreted galectin-3 is elevated in islets of obese/diabetic mice and humans, binding the calcium channel auxiliary subunit CACNG1 on β-cell membranes, inhibiting calcium influx and rapidly blocking glucose-stimulated insulin secretion [88]. In obesity-related low-grade inflammation, macrophage activation and infiltration link IR and β-cell dysfunction. High-fat/high-sugar diets significantly activate the transcriptional co-regulator CREBZF in macrophages, enhancing NF-κB signaling and pro-inflammatory cytokine expression, exacerbating IR and T2DM. Macrophage-specific CREBZF deficiency reduced adipose inflammation and improved systemic insulin sensitivity; co-culture experiments showed conditioned media from CREBZF-deficient macrophages enhanced adipocyte insulin response. Mechanistically, CREBZF competitively inhibits IκBα binding to p65, prolonging nuclear NF-κB retention and enhancing its transcriptional activity, leading to sustained cytokine expression [89].

In summary, these studies indicate systemic inflammation in high-fat/obese contexts further damages islet β-cells or exacerbates IR. As MPs are key inducers of systemic inflammation, they may exacerbate diabetes via these mechanisms.

### 5.2. Adipose Tissue Inflammation and Dysfunction

Adipose tissue is not only an energy store but also an endocrine organ with immune and metabolic regulatory functions, where macrophage phenotype balance is crucial for metabolic homeostasis. Physiologically, adipose tissue is dominated by anti-inflammatory M2 macrophages secreting IL-10 and TGF-β, regulating adipocyte differentiation and lipid metabolism. MPs exposure disrupts this balance, inducing a shift to pro-inflammatory M1 macrophages, a key driver of adipose inflammation and IR.

Macrophage phenotype switching is often triggered by adipocyte dysfunction. Energy excess or MPs stimulation causes adipocyte hypertrophy, apoptosis, and abnormal lipolysis, releasing danger signals like saturated fatty acids and free cholesterol; these activate adipocytes via TLR4/NF-κB to secrete chemokines like MCP-1, recruiting circulating monocytes into adipose tissue. Monocytes differentiate into macrophages and, upon phagocytosing apoptotic adipocytes, polarize to M1. As apoptotic adipocytes increase, M1 macrophages form “crown-like structures” around necrotic cells, creating inflammatory foci that continuously release pro-inflammatory factors. Recent studies found diet-derived MPs preferentially accumulate in white adipose tissue of HFD-fed obese mice, significantly inhibiting β-adrenergic and fasting-induced lipolysis. MPs exposure reduced protein expression and phosphorylation of key lipases Adipose Triglyceride Lipase (ATGL) and Hormone-Sensitive Lipase (HSL), impairing lipolysis and causing subcutaneous adipocyte hypertrophy. MPs also induced gut inflammation and hepatic lipid deposition, suggesting it exacerbates systemic metabolic disorder by hindering adipose lipid mobilization. These results indicate MPs may promote obesity and metabolic disease by inhibiting lipolysis, promoting lipid accumulation and local inflammation, releasing danger molecules like saturated fatty acids and free cholesterol [90]. Saturated fatty acids act as endogenous danger signals in adipose inflammation, activating inflammation via TLR4/NF-κB. In adipocyte-macrophage co-cultures, palmitate as a TLR4 ligand activates macrophage NF-κB, inducing adipocyte MCP-1 secretion and monocyte recruitment, establishing chronic inflammation [91]. Recent studies reveal adipocyte-derived microvesicles (ADMs) are key in macrophage polarization. ADMs from obese mice are rich in miR-155, which inhibits SOCS1 and activates STAT1, inducing M1 polarization; this in turn feeds back to adipocytes via M1-secreted factors, interfering with insulin signaling and glucose uptake, forming a vicious cycle [92]. Macrophage clearance efficiency of dead adipocytes is significantly affected by cell size. Adipocytes typically exceed 25 μm in diameter, beyond the physical threshold for classic macrophage clearance, leading to accumulation of dead cell debris. This debris undergoes secondary necrosis, triggering local pro-inflammatory responses even in lean, healthy animals. Laser-induced single adipocyte death confirmed adipocyte death alone is sufficient to induce crown-like structure formation, metabolic activation, and inflammatory macrophage phenotypes, indicating adipocyte size is a key driver of chronic adipose inflammation [93].

Kou et al. provide direct evidence linking MPs-induced adipose inflammation to metabolic disorder. MPs exposure caused gut dysbiosis, increasing lipopolysaccharide-producing pro-inflammatory bacteria like Desulfovibrionaceae, exacerbating systemic and local low-grade inflammation. Although macrophage infiltration was not directly measured, elevated adipose pro-inflammatory cytokines, activated lipogenesis pathways, and widespread suppression of thermogenesis reveal a macrophage-driven metabolic disorder mechanism. Thus, gut dysbiosis-exacerbated systemic inflammation likely promotes macrophage recruitment and activation in white and brown adipose, releasing TNF-α, IL-1β, etc., which inhibit insulin signaling, induce IR, promote preadipocyte differentiation and lipogenesis, increase fat accumulation, and suppress UCP1 and energy expenditure. This study supports MPs inducing systemic and adipose inflammation via gut dysbiosis, leading to increased lipogenesis, lipid accumulation, IR, and impaired thermogenesis through macrophage activation [94].

In summary, MPs, either directly or indirectly via gut microbiota, trigger and maintain a chronic adipose inflammatory state centered on M1 macrophages. This state promotes obesity and metabolic disease by inhibiting lipolysis, promoting lipogenesis, inducing IR, and suppressing thermogenesis.

### 5.3. Skeletal Muscle Insulin Resistance

Skeletal muscle is the primary site for insulin-mediated glucose uptake, accounting for 25–45% of postprandial glucose disposal at rest and up to 85% under insulin stimulation, making its insulin sensitivity crucial for glucose homeostasis. Aberrant macrophage activation here is often overlooked. Unlike pancreas and fat, skeletal muscle macrophages are primarily embryo-derived residents expressing markers like Lymphatic Vessel Endothelial Receptor-1 (LYVE1) and T cell immunoglobulin and mucin domain containing 4 (TIMD4), involved in muscle repair and metabolic regulation under physiological conditions but becoming dysfunctional under systemic inflammatory signals.

Skeletal muscle macrophage regulation of insulin sensitivity relies largely on endothelial-macrophage crosstalk. MPs-induced systemic inflammation reduces endothelial glucose transporter 1 (GLUT1) expression in muscle vasculature, triggering endothelial metabolic reprogramming and increased osteopontin secretion. Osteopontin, as a key vascular factor, directly activates perivascular resident macrophages, promoting their proliferation and activation. These macrophages secrete IL-6, inhibiting muscle cell insulin signaling—reducing Akt and TBC1D4 phosphorylation, hindering GLUT4 translocation, and decreasing insulin-stimulated glucose uptake. Activation of resident muscle macrophages is locally autonomous; depleting peripheral monocytes does not affect endothelial GLUT1 loss-induced macrophage proliferation and IR, indicating dependence on local microenvironment signals [95].

In chronic inflammation, M1 macrophages also contribute to low-grade muscle inflammation, inhibiting myofiber energy metabolism and repair, further reducing insulin sensitivity. MPs-induced systemic inflammation, initiated via the gut–liver axis, triggers synergistic activation and functional remodeling of macrophages in pancreas, adipose, and skeletal muscle. These organ-specific macrophages amplify inflammatory signals in concert, impairing β-cell function, driving adipose inflammation, and reducing muscle insulin sensitivity, collectively promoting metabolic disorder and forming a key pathological bridge between systemic inflammation and diabetes.

### 5.4. Microplastics and the Brain

Recent investigations have elucidated multifaceted mechanisms through which MPs induce metabolic dysregulation, with a pivotal role emerging for CNS-mediated pathways. Wang et al. demonstrated that exposure to low-density PE-MPs and their oxidized derivatives significantly induces oxidative stress, neuroinflammation, and BBB disruption in the murine cerebral cortex and hippocampus. This is accompanied by downregulation of key genes governing cholinergic synaptic signaling (e.g., Chat, Slc18a3, Slc5a7), culminating in reduced acetylcholine (ACh) levels. Beyond its canonical role in cognitive function, the cholinergic system modulates peripheral metabolism via vagal efferent pathways; its disruption therefore implicates a central mechanism by which MPs may perturb energy homeostasis. Although direct assessment of glycemic or insulinemic parameters was not performed, the marked elevation of pro-inflammatory cytokines (TNF-α, IL-1β, IL-6) within the brain parenchyma provides indirect evidence for the establishment of central IR. Established literature indicates that CNS neuroinflammation can disrupt insulin signaling through activation of the hypothalamic IKKβ/NF-κB pathway, thereby inducing central IR and perturbing systemic energy balance [96].

Complementing these findings, Lee et al. extended the investigation to an obesogenic context, revealing that PS-MP exposure exacerbates HFD-induced glucose intolerance and IR (elevated HOMA-IR), effects independent of body weight changes. This dissociation from adiposity suggests a direct, obesity-independent interference with glucose regulation, potentially via central mechanisms. The study demonstrated that circulating PS-MPs are extensively captured by immune cells, notably CD8^+^ T cells and monocytes, and promote the expansion and tissue infiltration of Ly6C^high^ inflammatory monocytes, including migration into the CNS. Within the hypothalamic arcuate nucleus (ARC), PS-MP deposition was concomitant with microglial activation, suggesting that peripherally activated inflammatory cells may traverse a compromised BBB, establishing a “peripheral-central inflammatory circuit” that amplifies hypothalamic inflammation and IR [10].

Notably, the ARC constitutes a critical nodal point for the homeostatic regulation of appetite and foraging behavior, and its neuronal function is highly susceptible to the surrounding inflammatory milieu. PS-MP-induced microglial activation and the consequent release of inflammatory mediators within the ARC are poised to directly impair the responsiveness of key neuronal populations (e.g., pro-opiomelanocortin [POMC] and agouti-related peptide [AgRP] neurons) to peripheral metabolic cues such as leptin, insulin, and glucose. Such impairment would predictably disrupt the intricate balance between satiety and hunger signaling, leading to maladaptive alterations in foraging behavior and energy intake patterns. While dynamic assessments of feeding behavior remain to be directly quantified in the context of MP exposure, the documented hypothalamic deposition and resultant neuroinflammation provide a compelling rationale for positing that chronic MP exposure contributes to metabolic dysregulation through the disruption of central appetitive networks.

Collectively, these findings advance a model wherein MPs not only instigate systemic inflammation via peripheral immune activation but also exert direct central effects. By infiltrating the CNS, MPs activate microglia, compromise BBB integrity, and interfere with cholinergic signaling. Through the resultant peripheral-central inflammatory axis, they induce hypothalamic dysfunction, ultimately contributing to central IR, impaired glucose homeostasis, and altered foraging behavior. This emerging pathway underscores a previously underappreciated neurotoxicological dimension of microplastic pollution with profound implications for metabolic health.

## 6. Controversies, Challenges, and Future Perspectives

### 6.1. Core Controversies

The emerging research linking microplastic exposure to diabetes faces a fundamental obstacle: profound heterogeneity in how studies are designed and executed. Studies vary widely in particle characteristics—such as polymer type, size, and shape. Differences in concentration, exposure duration, model organisms, and measured outcomes further complicate comparisons. As a result, quantitative synthesis of findings remains a major challenge [28,97]. This methodological fragmentation is more than a technical hurdle; it lies at the heart of ongoing debates over whether these pervasive pollutants pose a metabolic threat.

Compounding this challenge is growing recognition that dose–response relationships are often non-linear. Contrary to classical monotonic assumptions, several investigations point to inverted U-shaped or threshold effects [27]. For example, certain inflammatory markers may spike at intermediate exposure levels but paradoxically fall at higher concentrations—possibly reflecting cytotoxicity that suppresses overall function or the engagement of strong negative feedback loops. Such non-linear dynamics upend conventional risk-assessment frameworks, severely complicating efforts to extrapolate findings to environmentally relevant doses.

At the population level, a fundamental critical gap persists: while MPs have been detected in human feces, blood, placenta, and even breast milk---unequivocally confirming exposure---this evidence alone cannot establish a causal link to diabetes. There are currently no longitudinal human studies demonstrating that microplastic exposure leads to the development of T2DM. Animal studies offer important mechanistic insights but provide limited guidance for human risk assessment, as experimental doses often far exceed realistic human intake, and cross-species differences in toxicokinetics remain largely unexplored [23]. Yet the sobering projection that a 70-year-old adult may accumulate up to 5.01 × 10^4^ particles in their tissues [33] suggests that chronic, low-level exposure could eventually overwhelm homeostatic capacity—a prospect warranting serious concern about long-term risks.

Particle characteristics add another layer of complexity. NPs, owing to their minute size, more readily cross biological barriers; fibrous particles may inflict damage through distinct physical mechanisms. Moreover, the dominant molecular pathways engaged appear polymer-dependent: PS particles tend to activate the TLR4/NF-κB inflammatory cascade, whereas PVC disrupts PI3K/Akt signaling [98]. To date, systematic comparative studies across these variables are lacking, obscuring which particle traits most strongly drive metabolic disruption.

Finally, the dimension of combined exposure remains largely unaccounted for. MPs can act as vectors for environmental pollutants, forming complex mixtures with heavy metals and organic contaminants. Co-exposure to PS-MPs and Cu^2+^, for instance, potentiates hepatic injury in fish, yet whether such vector effects contribute to human diabetes pathogenesis is unknown. Existing human evidence is predominantly cross-sectional—for example, associations between urinary MP levels and hyperglycemia—and lacks the longitudinal rigor needed for causal validation. Given the intricate web of human metabolic regulation, the evidentiary chain for MPs as a direct diabetogenic agent remains incomplete. In short, the field is shifting from merely documenting exposure to grappling with the challenge of confirming pathogenicity. Closing this gap will require coordinated efforts: methodological standardization, robust cross-species dosimetric models, and prospective cohort studies designed to rigorously test the causal hypothesis.

### 6.2. Future Research Directions

Technical integration and mechanistic dissection: Develop gut–liver axis organ-on-a-chip models integrating multi-omics data (transcriptomics: TLR4/NF-κB pathway genes; metabolomics: short-chain fatty acids, glucose metabolites; proteomics: cytokines, tight junction proteins) to build toxicity prediction models; dissect spatiotemporal dynamics of gut microbiota-macrophage crosstalk; clarify MPs distribution and immune responses along intestinal segments.

Dose modeling and human studies: Establish cross-species dose conversion models based on body surface area, optimized by MP physicochemical properties (specific surface area, surface charge); develop long-term low-dose animal exposure models (≥6 months) mimicking real human exposure; conduct multicenter cohort studies screening high-risk individuals (BMI ≥ 25, family history of diabetes), monitoring dynamic associations between blood/urine MPs concentrations and diabetes incidence.

Intervention strategies: Develop MPs clearance agents, e.g., probiotics like *Lacticaseibacillus paracasei DT66*, *Lactiplantibacillus plantarum DT88*, which adsorb MPs, promote excretion, and alleviate gut inflammation [99]. Target inflammatory pathway inhibitors: TLR4 inhibitor TAK-242, MyD88 inhibitor TJ-M2010-5, VDR agonist calcipotriol, to block MPs-induced inflammatory cascades [100,101,102,103]. Explore combination therapies (probiotics + inhibitors) for dual gut clearance and systemic anti-inflammatory action to improve metabolic disorder.

The escalating contamination of the environment by microplastics and nanoplastics (MNPs) represents a pressing public health concern, yet traditional toxicological models inadequately capture the complex human tissue microenvironments and immune-metabolic crosstalk essential for understanding MNP pathogenesis. Organoid technology has emerged as a transformative platform, offering physiologically relevant, human-specific insights that transcend the inherent constraints of conventional systems [104].

Recent advances within the gut–liver axis framework have yielded critical mechanistic discoveries. Intestinal organoids have revealed that PS-NPs exhibit selective tropism for enteroendocrine cells, where accumulation triggers inflammatory responses and apoptotic pathways—findings with direct implications for metabolic signaling. Building upon these insights, an integrated “gut–liver-pancreas” organ-on-a-chip platform would enable comprehensive recapitulation of the dynamic toxicity cascade following oral MNP exposure: from initial intestinal barrier disruption, through hepatic inflammatory amplification, to culminating pancreatic islet dysfunction.

To improve translational relevance, the field should prioritize two complementary goals. First, we need chronic, low-dose exposure models that reflect real-world human exposure, rather than relying on acute high-dose paradigms. Second, we must develop multi-organ microphysiological systems that include vascular and immune components. These platforms will be essential for understanding the systemic effects of micro- and nanoplastic bioaccumulation. Such next-generation platforms promise to transform our mechanistic understanding of environment-induced metabolic diseases and provide robust frameworks for evidence-based risk assessment.

## 7. Conclusions

MPs have emerged as pervasive environmental contaminants, leading to chronic human exposure through ingestion, inhalation, and dermal contact. Based on a large body of evidence from animal and in vitro models, the gastrointestinal tract is proposed to serve as the primary site of action, where MPs may initiate a pathological cascade via the gut–liver axis. Current evidence suggests this process involves the modulation of macrophage M1 polarization, disruption of intestinal barrier integrity, and induction of gut microbiota dysbiosis. These local insults are hypothesized to propagate hepatic inflammation, which could subsequently extend to peripheral metabolic tissues—including the pancreas, adipose tissue, and skeletal muscle—potentially culminating in multi-organ IR and impaired β-cell function. While this cascade provides a compelling and biologically plausible mechanism, it is crucial to acknowledge that these pathways have yet to be definitively validated in human populations. Critically, IR is not only the central pathophysiological mechanism underlying T2DM but also a key pre-disease state amenable to early identification and intervention. This state can persist for years before the clinical onset of T2DM and its complications, presenting a crucial window for mitigating environmental risk factors. Elucidating the role of microplastic exposure in the development of IR is therefore of paramount importance for the primary prevention of T2DM.

Despite this compelling link, several critical questions remain unresolved, including dose–response relationships, particle-specific effects (considering size, polymer type, and shape), the impact of complex environmental mixtures, and the need for robust human validation. Future research must leverage advanced methodologies—such as multi-omics integration, organ-on-a-chip platforms, long-term low-dose exposure models, and prospective cohort studies—to deepen our mechanistic understanding and facilitate clinical translation. Incorporating microplastic exposure into metabolic disease risk assessment frameworks offers a novel perspective for the precision prevention of environment-related diabetes. From a broader public health standpoint, these findings underscore the urgent need for global cooperation to curb plastic production, develop sustainable alternatives, improve waste management, and devise innovative interventions (e.g., specific probiotics or adsorbents) that can effectively block the absorption and accumulation of MPs in the body. Such multifaceted strategies are essential to reduce population-level exposure at its source and ultimately safeguard public health.

## Figures and Tables

**Table 1:** Mechanisms of microplastic-induced macrophage polarization and dysfunction in metabolic organs.

Organ/Tissue	Macrophage Subtype/Origin	Primary Activation Pathways	Key Signaling Pathways	Effector Inflammatory Factors	Pathophysiological Consequences
**Intestine**	Intestinal resident macrophages	Direct phagocytosis of MPs; gut microbiota dysbiosis (LPS release)	TLR4/NF-κB	TNF-α, IL-1β, IL-6	Downregulation of tight junction proteins (ZO-1, occludin); reduced mucus secretion; increased intestinal permeability (“leaky gut”)
**Liver**	Kupffer cells (resident macrophages)	Phagocytosis of MPs; LPS influx via portal vein	TLR4/NF-κB/NLRP3	TNF-α, IL-1β, IL-6	Hepatic insulin resistance (inhibition of IRS1/Akt signaling); synthesis and release of acute-phase proteins (CRP, SAA)
**Adipose tissue**	Adipose tissue macrophages (ATMs)	Saturated fatty acids (e.g., palmitate); adipocyte-derived exosomes (miR-155)	TLR4/NF-κB	TNF-α, IL-1β, MCP-1	Crown-like structure formation; suppressed lipolysis (ATGL/HSL↓); impaired thermogenesis (UCP1↓)
**Pancreas**	Islet-resident macrophages	Systemic inflammatory factors; M1 macrophage-derived exosomes (miR-212-5p)	NF-κB; TLR4	IL-1β, TNF-α	Downregulation of β-cell functional genes (Pdx1, Ins1/2); impaired glucose-stimulated insulin secretion
**Skeletal muscle**	Resident macrophages (LYVE1^+^, TIMD4^+^)	Endothelium-derived osteopontin (OPN)	Local IL-6 paracrine signaling	IL-6	Reduced phosphorylation of AKT/TBC1D4; impaired GLUT4 translocation; decreased insulin-stimulated glucose uptake
**Brain (hypothalamus)**	Microglia	MPs crossing the blood–brain barrier; infiltration of peripheral inflammatory monocytes	NF-κB	TNF-α, IL-1β, IL-6	Microglial activation; hypothalamic insulin resistance; dysfunction of POMC/AgRP neurons, leading to dysregulated appetite and energy balance

## Data Availability

Data availability is not applicable to this article as no new data were created or analyzed in this study.

## List of Abbreviations

MPs	Microplastics
NPs	Nanoplastics
T2DM	Type 2 Diabetes Mellitus
PP	Polypropylene
PS	Polystyrene
PET	Polyethylene Terephthalate
PVC	Polyvinyl Chloride
PE	Polyethylene
PUR	Polyurethane
ROS	Reactive Oxygen Species
IR	Insulin Resistance
VDR	Vitamin D Receptor
GST	Glutathione S-transferase
CAT	Catalase
GR	Glutathione Reductase
iPSC	Induced Pluripotent Stem Cells
Heps	Hepatocytes
HFD	High-Fat Diet
ATGL	Adipose Triglyceride Lipase
HSL	Hormone-Sensitive Lipase
ALP	Alkaline phosphatase
γ-GGT	Gamma-Glutamyl Transferase
PGLYRPs	Peptidoglycan recognition proteins
SDH	Succinate dehydrogenase
TEER	Trans-Endothelial/Trans-Epithelial Electrical Resistance
Cdx2	Caudal-type homeobox transcription factor 2
MLCK	Myosin light-chain kinase
SOD	Superoxide dismutase
GSH-Px	Glutathione peroxidase
MET	Macrophage extracellular trap
GMTriP-K	Lysine N-acetylglucosamine N-acetylmuramic tripeptide
NOD2	Nucleotide-binding oligomerization domain 2
HIF-1α	Hypoxia inducible factor 1 subunit alpha
IGFBP7	Insulin-like growth factor-binding protein 7
SAA	Serum amyloid A
ADMs	Adipocyte-derived microvesicles
GLUT1	Glucose transporter 1
LYVE1	Lymphatic Vessel Endothelial Receptor-1
TIMD4	T cell immunoglobulin and mucin domain containing 4
CNS	Central Nervous System
BBB	Blood–brain barrier
ACh	acetylcholine
ARC	Arcuate nucleus
POMC	Pro-opiomelanocortin
AgRP	Agouti-related peptide
MNPs	Microplastics and nanoplastics
